# Characteristics and driving factors of spatiotemporal changes in soil erosion in the karst plateau mountainous region over 20 years

**DOI:** 10.1371/journal.pone.0314266

**Published:** 2024-11-21

**Authors:** Yi Bai, Yiyang Zhang, Sujin Zhang, Jianfeng Wu, Xiaoqing Zhao, Fei Zhao

**Affiliations:** 1 School of Geography and Resources, Guizhou Education University, Guiyang, Guizhou, China; 2 Guizhou Provincial Key Laboratory of Geographic State Monitoring of Watershed, Guiyang, Guizhou, China; 3 School of Earth Sciences, Yunnan University, Kunming, China; Western Carolina University, UNITED STATES OF AMERICA

## Abstract

Soil erosion is one of the main issues that endangers global ecosystems. This study explored the spatiotemporal distribution of soil erosion and its drivers in the karst plateau mountainous region. A detailed examination of topography, soil, vegetation, land use, and precipitation data from 2000 to 2020 was conducted in Bijie City using the revised universal soil loss equation model. We also explored the driving forces using a geographical detector. The findings show that between 2000 and 2020, soil erosion first decreased, followed by an increase. The southwest, south, and northern regions contained the highest intensity of soil erosion. Land use, slope, and precipitation are the primary factors influencing soil erosion, with slopes having the greatest impact. By improving our understanding of the dynamics of soil erosion and the primary variables that influence it in karst plateau mountainous environments, our findings can assist in the development of strategies and technical support for sustainable soil and water conservation.

## Introduction

Soil erosion is a serious ecological hazard that causes severe environmental deterioration at the global scale [1]. As well as causing economic losses to agricultural production, soil erosion also irreversibly affects biodiversity [2] and ecological balance [3]. Severe risks associated with soil erosion include the depletion of land resources [4], loss of soil fertility [5], exacerbation of droughts or floods [6], and reduced agricultural output [7], leading to international attention. In the karst plateau mountainous regions, the ecological environment is particularly vulnerable because of its unique geological situation. Soil generation processes in this zone are slow, and problems such as soil erosion and land degradation are serious [8,9]. The district has a mild climate, favorable water and heat conditions, and high vegetation cover, but it is an ecologically fragile area owing to insufficient soil-forming materials, poor vegetation standing conditions, high ecological sensitivity, and prominent human–land conflicts [10]. Therefore, studies on the features and contributing variables of spatiotemporal changes in the karst plateau mountainous regions are crucial for developing strategies for land management, improving agricultural sustainability, and advancing regional development.

Research on soil erosion has considerably advanced, especially through empirical modeling of soil erosion distribution [11–15]. However, assessment models are often hampered by poor data quality and architectural limitations, particularly when predicting soil risk over a broad spatial scale [16]. In contrast, the two most widely used models are the universal soil loss equation (USLE) and revised USLE (RUSLE) models [17,18]. The RUSLE model is based on experimental observations and combines statistical analyses to quantify soil erosion factors [19]. RUSLE incorporates various factors that affect erosion, including runoff and rainfall, soil erodibility, slope characteristics, vegetation cover, and the effectiveness of erosion control techniques. Da Cunha et al [20] used the RUSLE model to assess soil loss in the Indaiá watershed in Brazil, emphasizing its widespread global application for soil loss estimation. Similarly, Al-Mamari et al [21] estimated annual soil erosion in the watershed upstream of the Assarin Dam in Oman, by integrating field surveys, RUSLE modeling, and remote sensing techniques. This technology has been successfully applied to soil erosion studies in several regions of China, including the mountainous areas of southern Yunnan, the Loess Plateau, the middle and lower reaches of the Yangtze River, and the karst region, yielding favorable results [22]. The RUSLE and geographic information science (GIS) techniques have been applied together to assess soil erosion in different watersheds worldwide. Chen et al [23] estimated the average annual rate of soil erosion in the Mawo Mountain Karst Basin of northwestern Guizhou from 1980 to 2000 using the RUSLE model and analyzed the sensitivity of each sub-model. Wang et al [24] employed the GIS and RUSLE models, along with the geodetector method, to study the quantitative attribution of soil erosion in karst areas with different geomorphological patterns in the Sancha River Basin. Many scholars have also combined RUSLE and GIS technologies for monitoring and assessing soil erosion and drought in different regions [25–28].

The fundamental attributes of soil erosion are reflected in spatiotemporal dynamics. Yu et al studied the dynamics of slope-cultivated areas across various topographical gradients using high-definition remote sensing imagery from 2004 to 2020 [9]. Currently, soil erosion research is paying increasing attention to multiscale assessment and scale effect analysis, focusing on the uncertainty analysis triggered by the scale change of the influencing factor [29]. Emphasizing spatiotemporal dimensions is an essential direction in the current study [30]. Fang et al [31] evaluated soil and water conservation in Guizhou Province using the RUSLE model from 2000 to 2019. In addition to studying spatiotemporal characteristics, exploring the driving factors is a key part of soil erosion research. Matomela et al [32] developed an innovative method by merging the SDR, InVEST, and geographical detector models to examine spatiotemporal changes and identify the major factors that influence erosion [32]. In a study focusing on representative karst basins with diverse geomorphic features, Wang et al [33] used the RUSLE model and a geographical detector. Two approaches are often used to determine the causes of soil erosion: non-spatial and spatial modeling [34]. Non-spatial modeling methods include, but are not limited to, linear regression [10] and redundancy analyses [35]. Spatial modeling encompasses techniques such as geographical detector [36] and morphometric analysis [14]. As spatial models fully consider the spatial heterogeneity of drivers, geographical detectors have analytical advantages over traditional statistical analysis methods [37].

Despite these advancements, several research gaps remain. Most of the current research focuses on assessing soil erosion at a single scale, lacking multiscale spatiotemporal data analysis. Furthermore, studies on the integrated assessment of drivers and their interactions are inadequate or uncertainty persists in studies on current models on small-scale soil erosion changes. Conducting small-scale research can assist policymakers in gaining insights into the current state of soil erosion in specific areas and develop strategies tailored to local conditions. Guizhou Province, where Bijie City is located, has numerous karst landscapes. Although a majority of the research has focused on landslides [38,39] and other geological hazards, limited attention has been paid to temporal variations in soil erosion. Given that soil erosion can lead to degradation, increased runoff, and geological disasters such as mudslides and landslides [40], it is crucial to investigate changes in soil erosion in Bijie.

The primary objective of this study is to examine the spatiotemporal characteristics of soil erosion and its contributing factors. The theoretical framework is based on soil erosion dynamics and ecosystem service theory. We hypothesize that the main drivers of soil erosion include precipitation, topography, soil properties, and land use practices. The interaction of these factors at different spatiotemporal scales determines the pattern and intensity of soil erosion.

Initially, we conducted a comprehensive analysis of precipitation data, soil properties, and remote sensing images of the watershed. Using the RUSLE model, we investigated the varying characteristics and trends of soil erosion in the research area. Subsequently, we employed a geographical detector approach to assess the impacts of variables such as vegetation cover and precipitation on soil erosion features and their interactions. A key innovation of this study is the utilization of multiscale spatiotemporal data, enhancing the precision and confidence of our findings. In addition, this study explored the significance of important variables and discussed the characteristics of soil erosion under different slope conditions in detail. These findings suggest the need for specific management and ecological restoration programs by local governments and land administrators to reduce soil erosion and promote sustainable regional development.

## Materials and methods

### Study area

**Fig 1:**
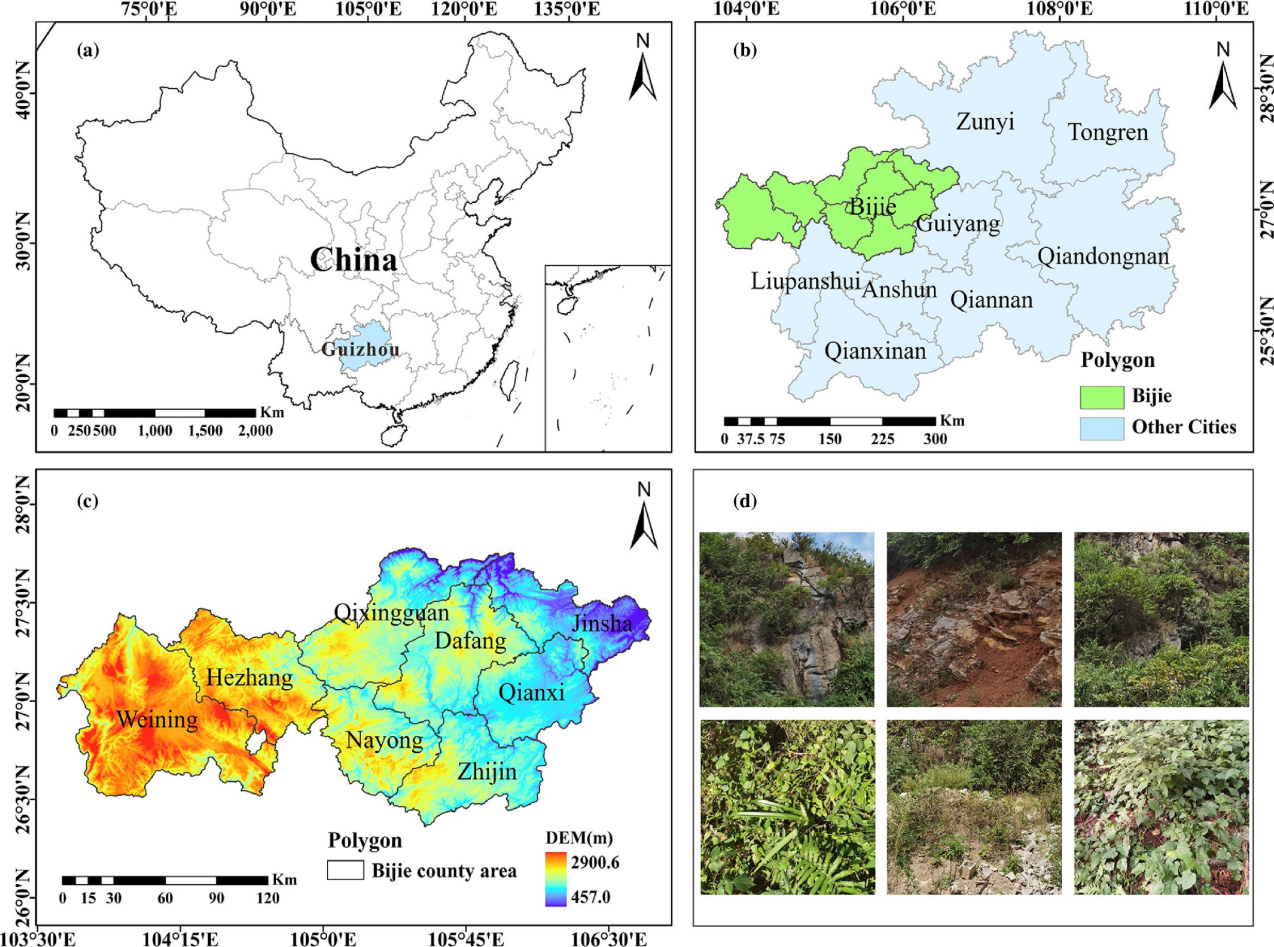
Overview of study area. (a) Location of Guizhou in China. (b) Location of the study area in Guizhou. (c) County subdivisions. (d) Soil erosion landform.

Bijie is situated on the Guizhou Plateau, a typical karst plateau mountain region (105°36′–106°43′E, 26°21′–27°46′N) (Fig 1). Soil erosion and land degradation caused by karst landscapes in the region have led to ecological degradation and poor production conditions, which have in turn led to a series of socioeconomic problems, including poverty and poor transportation.

The region has a complex geological structure characterized by the interplay of folds and fractures, showing a diverse karst topography, with the terrain progressively dropping from the west to the east. The average elevation reaches 1400 m, with the highest point at 2,900.6 m above sea level and the lowest point at 457 m. The average annual temperature is 14.6°C, and the average annual precipitation is 1,047.7 mm, indicative of a temperate climate. In the past, owing to long-term irrational utilization, a large amount of native vegetation has been destroyed, leading to increased soil erosion. In June 1988, Bijie launched a pilot area project for “development and poverty alleviation and ecological construction” [41], focusing on comprehensive soil erosion management and adopting a number of soil and water conservation strategies. These efforts have improved local soil erosion to a certain extent [42].

### Data sources and processing

The digital elevation model data were obtained from the Geospatial Data Cloud. A total of 60 months of precipitation data are represented by 1-km month-by-month data for five periods: 2000, 2005, 2010, 2015, and 2020 from the National Earth System Science Data Center. The vegetation cover data were provided by the maximum 30 m annual normalized difference vegetation index (NDVI) dataset in China [43]. The Harmonized World Soil Database provided clay content, bulk weight, depth, and organic matter content of the soil [42]. The GLC_FCS30 dataset was used for land-use data with a fine-grained classification system [44]. The sources, resolutions, and citations of all data are detailed in Table 1. Preprocessing of the data, including mosaicking, extraction by a mask, coordinate conversion, and resampling, was performed using ArcGIS 10.5, and a 30 m × 30 m raster image was resampled.

**Table 1:** Data type, resolution, and source.

Type	Resolution		Source
	Spatial	Temporal	
**Digital elevation model**	30 m	/	https://www.gscloud.cn/
**Precipitation dataset**	1 km	Monthly	http://www.geodata.cn
**Annual maximum NDVI dataset**	30 m	Annual	http://www.nesdc.org.cn/
**Soil data**	1 km	/	https://data.tpdc.ac.cn/
**Land-use type data (GLS-FCS30)**	30 m	Annual	https://data.casearth.cn/

**Fig 2:**
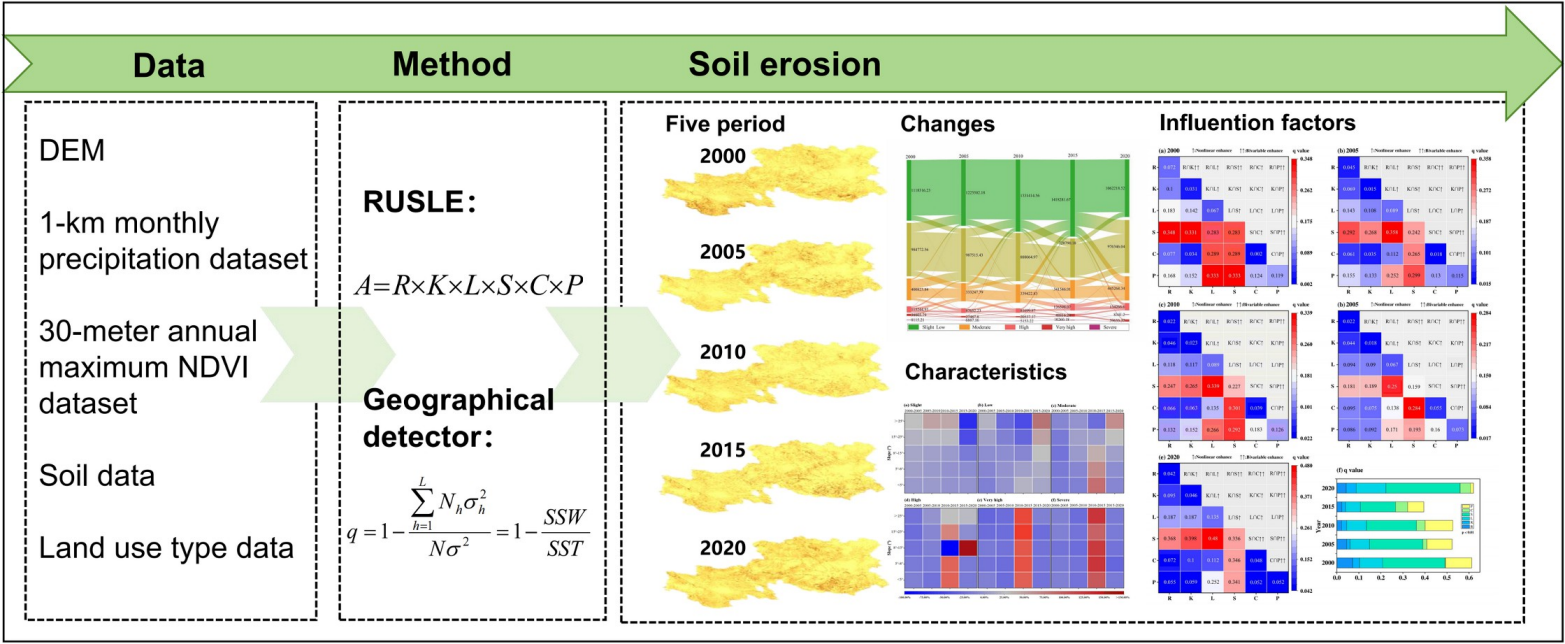
Methodology flow chart.

Using the available data and RUSLE model, this study examined the spatiotemporal distribution patterns, as well as the evolutionary aspects of soil erosion. This study also examined the use of geographical detection approaches to analyze the factors that contribute to soil erosion disasters. The research concept for this study is illustrated in Fig 2.

### RUSLE model

The relationship between the main contributing elements and slope erosion can be quantitatively characterized using the RUSLE model [18]. The expression is:

A=R×K×L×S×C×P (1)

where *A* (*t·ha*^*−1·*^*a*^*−1*^) denotes the average annual soil loss per unit area for any section of sloping cropland, the intensity of soil erosion can be classified into six classes based on the A-value. This equation incorporates six critical factors: the precipitation erosivity (*R*), soil erodibility (*K*), slope length (*L*), slope steepness (*S*), soil and water conservation measures (*P*), and vegetation cover (*C*). Using the ArcGIS and RUSLE models, six parameters were raster multiplied to determine the *A*-values in the research region. Among these, *K*, *L*, *S*, *P* are all relatively static over time, whereas *R* [45] and *C* [46] are analyzed on a monthly or seasonal basis. Consequently, exploring the interplay among precipitation, vegetation, and soil erosion on an image metric scale is crucial [47]. The magnitude and importance of the applicable thresholds are specified in the individual sections.

1. The amount, composition, intensity, speed, and diameter of raindrops affect factor *R* [48]. Wischmeier proposed a basic approach for determining rainfall erosion rate [18]. Subsequently, researchers have attempted to construct various simplified models of rainfall erosivity, encompassing annual [49], seasonal [49], monthly [50], and daily [51] frameworks. After comprehensively comparing these models, an equation deemed appropriate for the climatic conditions of southern China was adopted to compute *R*, as shown in Eq (2) [52]:
$$R=\Sigma_{i-1}^{12}(-1.5527+{0.1792P}_{i})$$ (2)
  where *P*_*i*_ denotes the multiyear average monthly precipitation (mm).
2. *K* is a crucial determinant of soil erodibility and measures susceptibility to erosion. This measurement is intricately linked to environmental variables including rainfall, runoff, soil texture, and soil permeability. A higher *K* value indicates lower resistance to erosion. In this study, *K* was calculated using the soil erosion–productivity impact calculation model (EPIC) [53], as described in Eqs 3 and 4:
$$K_{EPIC}=\left\{0.2+0.3\;exp\;\left[-0.0256SAN\left(1-\frac{SIL}{100}\right)\right]\right\}\times {\left[\frac{SIL}{CLA+SIL}\right]}^{0.3}\times \left[1-\frac{0.25C}{C+exp(3.72-2.59C)}\right]\times \left[1-\frac{0.7(1-SAN)}{\left(1-SAN)+exp(22.9\times (1-SAN)-5.51\right)}\right]$$ (3)
$$K=\left(-0.01383+0.51575K_{EPIC}\right)\times 0.1317$$ (4)
  where *SIL* is the percentage of fines content, *CLA* is the percentage of clay content, *C* is the percentage of organic carbon, and *SAN* is the percentage of sand.
3. Factor *L* quantifies the soil erosion rate on a given slope length relative to that on a standardized plot, assuming identical conditions for rainfall, vegetation, soil, slope, and other surface variables. Similarly, *S* represents the ratio of erosion rates between a specific slope and a standardized plot under uniform conditions. This study used the Fu et al [54] analysis method to calculate *L* and *S* values, as described in Eqs 5 and 6:
$$L=\frac{\gamma_{i}^{m+1}-\gamma_{i-1}^{m+1}}{\left(\gamma_{i_{i}}-\gamma_{i-1_{i}}\right)\cdot {\left(22.13\right)}^{m}}\;m=\left\{\begin{array}{c} 0.2\;\theta \leq 0.5^{{}^{\circ}}\\ 0.3\;0.5^{{}^{\circ}}<\theta \leq 1.5^{{}^{\circ}}\\ 0.4\;1.5^{{}^{\circ}}<\theta \leq 3^{{}^{\circ}}\\ 0.5\;\theta >3^{{}^{\circ}} \end{array}\right.$$ (5)
$$S=\left\{\begin{array}{cc} 10.8\sin\theta +0.03 & \theta <5^{{}^{\circ}}\\ 16.8\sin\theta -0.5 & 5^{{}^{\circ}}\leq \theta <10^{{}^{\circ}}\\ 21.9\sin\theta -0.96 & \theta \geq 10^{{}^{\circ}} \end{array}\right.$$ (6)
  where *γ*_*i*_ and *γ*_*i*−1_ denote the slope lengths of the *i*th and (*i*−1)th slope segments; *θ* denotes the slope gradient; and *m* denotes the *L* index, which varies with *θ*.
4. By reducing the effects of runoff and boosting the natural resilience of the land to erosion, factor C is essential for minimizing soil erosion, with values ranging from 0 to 1, whereas excellent vegetation is indicated by a C value that is close to 0.
  The calculation model suggested by Cai et al [55] is expressed in Eqs 7 and 8:
$$F_{FVC}=\frac{NDVI-NDVI_{\min}}{NDVI_{\max}-NDVI_{\min}}$$ (7)
$$C=\left\{\begin{array}{cc} 1 & F_{FVC}=0\\ 0.6508-0.3436 & 0<F_{FVC}\leq 78.3\%\\ 0 & F_{FVC}\geq 78.3\% \end{array}\right.$$ (8)
  where *F*_*FVC*_ represents the amount of vegetation, NDVI represents the normalized vegetation cover index, *NDVI*_*min*_ denotes the cover index for bare or non-vegetated land, and *NDVI*_*max*_ indicates the cover index for a fully vegetated site.
5. *P* assesses the efficacy of soil erosion control measures, with values ranging from 0 to 1. A *P* value of 1 signifies that no soil protection measure has been implemented, whereas a *P* value of 0 indicates that the district has taken measures to prevent soil erosion. The land-use type is often used to determine *P* values [56]. In light of the specific situation in Bijie, different factors for soil conservation measures were assigned to various land-use types [57] (Table 2):

**Table 2:** *P* values for several categories of land usage.

Land use	Grassland	Forest	Paddy land	Water body	Construction land	Unused land
***P***	1	1	0.35	0	0	0.1

### Geographical detector

The set of statistical methods comprises a geographical detector. This study aimed to investigate the drivers behind spatial variability in environmental and socioeconomic datasets. In particular, the factor detector can evaluate how well each factor accounts for the spatial distribution, and the interaction detector can explore the interactions and joint effects among multiple factors. To examine how the six factors interact with soil erosion (A), we primarily used factor and interaction detectors.

1. Factor detector: This was employed to analyze whether the six influencing factors were the main causes of spatial variability in soil erosion. When variables and geographical changes exhibit a strong spatial association, the former influences the development of the latter. The q-value, which ranges from 0 to 1, denotes the outcome. Higher q values indicate that the influencing factors can better explain soil erosion.
2. Interaction detector: This detector can be used to assess how the combined effects of the constituents explain the dependent variable or establish whether the effects of these factors on A are independent.

## Results

### Variation of soil erosion parameters in space and time

**Fig 3:**
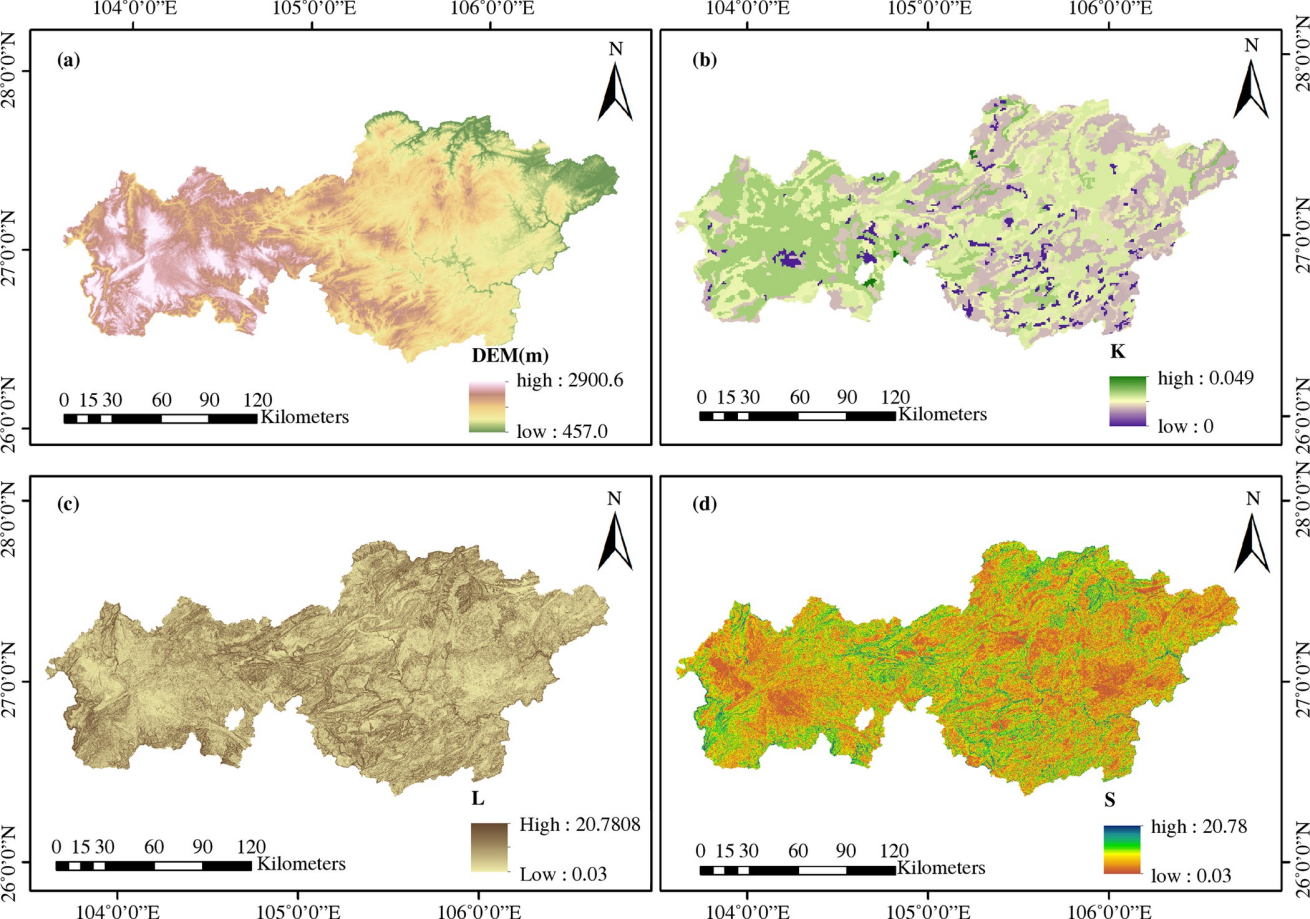
Digital elevation model and spatial distributions of *K*, *L*, and *S* values.

**Fig 4:**
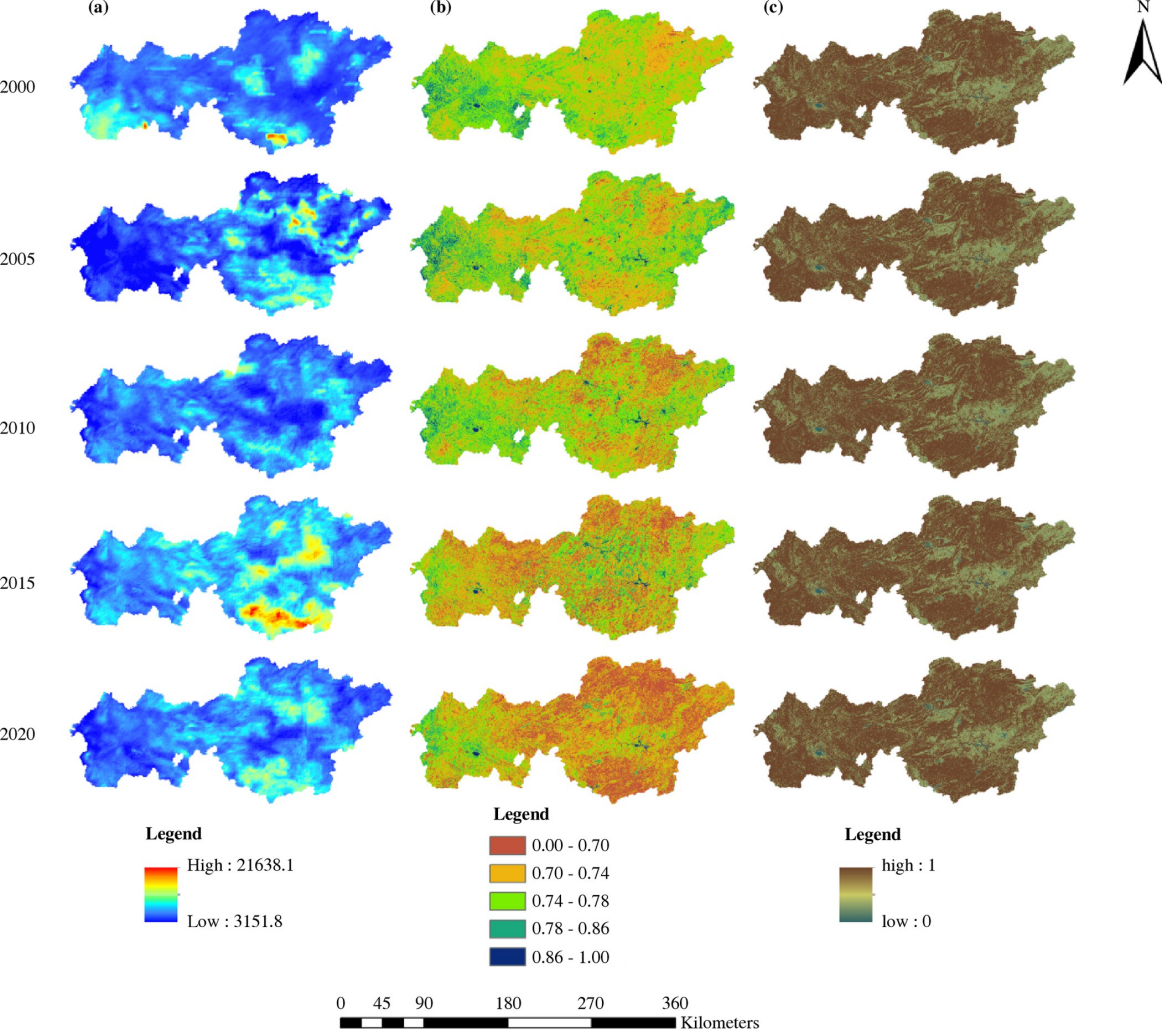
Spatiotemporal distributions. (a) *R*, (b) *C*, and (c) *P* values.

The results of the RUSLE model computations for various factors are shown in Figs 3 and 4. Fig 3A shows a gradual decline in the Bijie terrain from west to east. Based on Eqs 3 and 4, the calculated soil erodibility coefficient K values (Fig 3B) revealed significant spatial differences and clear clustering characteristics of K values in Bijie. Most sites with the lowest K values were located in the eastern section, whereas the western section contained a concentration of more erodible areas, indicating that the K value gradually increased from east to west. Based on Eqs 5 and 6, the spatial distributions of the L and S values are displayed after determining the corresponding values (Fig 3C and 3D). The S factor influences the surface runoff flow rate, infiltration rate, and soil stability, which, in turn, have an indirect impact on soil erosion. The L factor acts on the soil erosion process through mechanisms such as influencing the rainfall catchment and sediment transport paths on the slope. The central, northern, and southern portions of Bijie exhibited higher concentrations of the L and S factors, whereas the eastern and western regions exhibited a tendency toward decreasing values.

*R* is a key indicator for assessing the soil erosion potential and is crucial in soil and water conservation studies of fragile ecosystems in southwestern karst areas [58]. The *R* values for Bijie were calculated by combining the monthly precipitation data using Eq 2 (Fig 4A). Overall, the *R* value increased from west to east, peaking in the southeastern region. The spatial distributions of rainfall erosion in 2000, 2010, and 2020 were relatively balanced, whereas the eastern and southeastern regions accounted for the majority of rainfall erosion in both 2005 and 2015.

A decrease in vegetation cover factor *C* usually indicates a weakening of soil erosion. The spatiotemporal distribution of factor *C* calculated using Eqs 7 and 8 (Fig 4B) shows a decreasing trend with time, indicating that soil erosion worsened during the period. The data showed that when the value of *C* decreased, soil erosion typically increases. The distribution of *C* values in 2000, 2005, and 2010 revealed that the west had high values, whereas the east had low values. The distribution of the spatial heterogeneity of C values in 2015 and 2020 was more evident and manifested in high-value areas. Contraction primarily occurred in the western and central regions of Bijie.

By altering the water flow pattern, slope, and catchment course, soil and water conservation methods can significantly minimize runoff volume, flow rate, and flow-down soil erosion [59]. The mean values of *P* for the five periods in Bijie were 0.803, 0.803, 0.802, 0.801, and 0.799, respectively, indicating that the efficiency of the water and soil conservation methods steadily improved during the study. As Fig 4C illustrates, the results of the eastern region outperformed those of the western region, demonstrating that during the course of the five time periods, the *P* factor spatial arrangement remained consistent. The greatest benefits of soil and water conservation were observed in the east-central region.

### Spatiotemporal distributions of soil conservation

A detailed analysis of the dynamics of soil erosion can aid in identifying motivating elements. This provides an important theoretical basis for researchers and scientific guidance for policymakers to design effective soil erosion control measures and formulate relevant regulations [60]. Eq 1 was used to compute the A value for the research area. Six degrees of erosion were identified in the study region using Soil Erosion Classification and Grading Standard SL190-2007 (Table 3).

**Table 3:** Classification criteria for soil erosion intensity.

Classification	Grade	Soil erosion modulus (*t·ha*^*−1*^*·a*^*−1*^)
**1**	Slight	<5
**2**	Low	5–25
**3**	Moderate	25–50
**4**	High	50–80
**5**	Very high	80–150
**6**	Severe	>150

**Fig 5:**
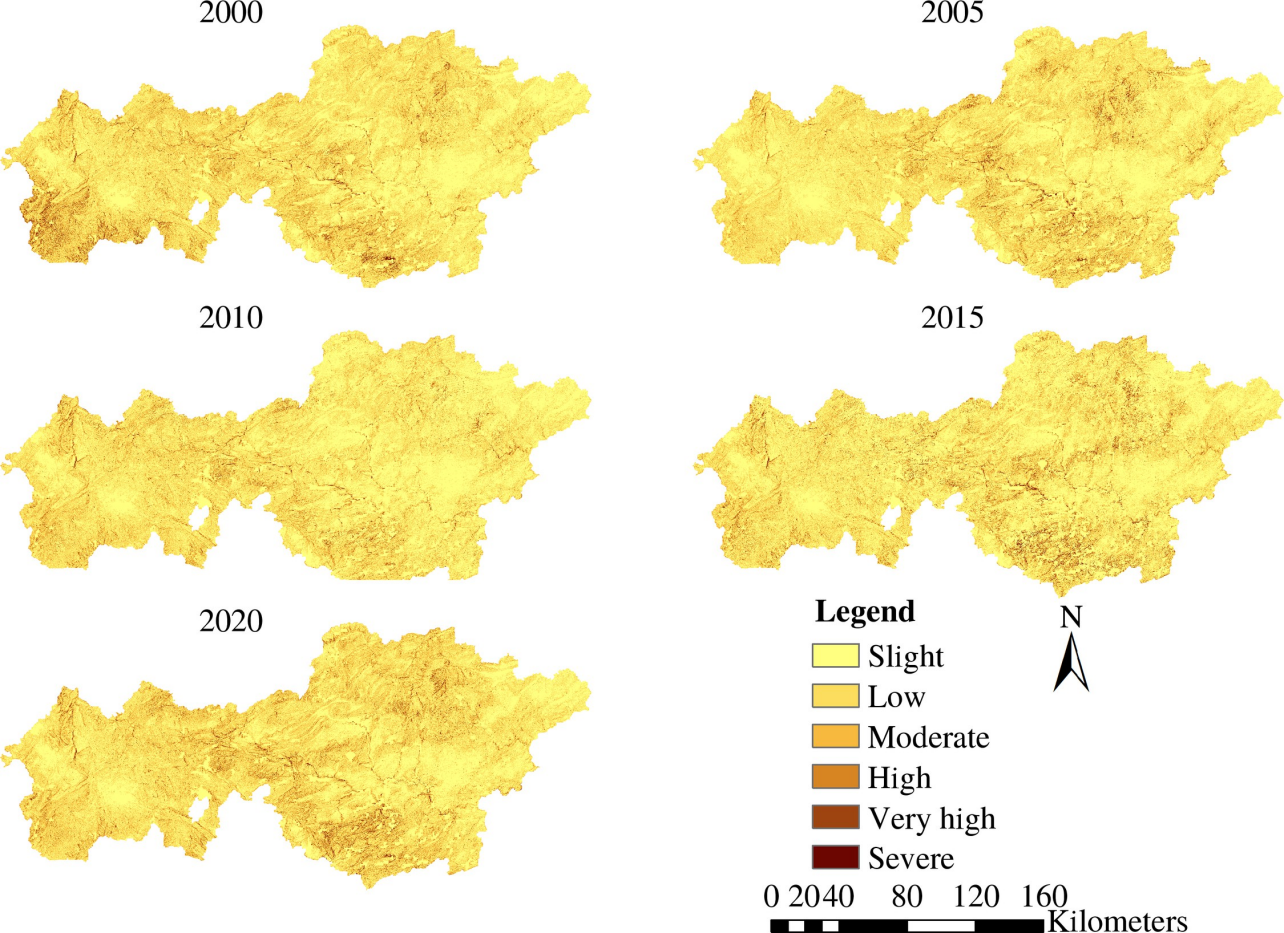
Spatial distribution of soil erosion in Bijie over the past 20 years.

Fig 5 illustrates that the research area experienced mild and slight soil erosion between 2000 and 2020 over 76% of the area. Spatially, soil erosion has evident differences in spatial distribution. Areas with moderate erosion levels and below were predominantly found in the central-western, east-central, and northeastern regions. In contrast, the southwest, south, and north are mostly located in regions with high levels of erosion. In western and southern Bijie, there was a discernible increase in the severity of soil erosion in 2000. A reduction in erosion intensity was observed in 2005 and 2010. This is similar to the findings of Zhang and Weng [61] and Xie et al [62], who implemented ecological engineering measures, such as converting ploughed land to forests and closing off mountains for reforestation, in certain Bijie experimental areas after 2000. However, a resurgence in severity occurred in 2015, with a focal concentration in the southern regions of the city. By 2020, the intensity of soil erosion had intensified, particularly in the south-central areas. There are limited studies on Bijie City covering the period from 2015 to 2020. However, Tian et al [63] noted a significant increase in the aggregation of arable land in Bijie City in 2018. They attributed this phenomenon to the promotion of converting farmland to forests and grasslands, which led to a reduction in arable land continuity and increased fragmentation and land density. Guo et al [64] observed that the area of severe rocky desertification in Bijie City was larger in 2015 compared to 2010 and 2020.

**Fig 6:**
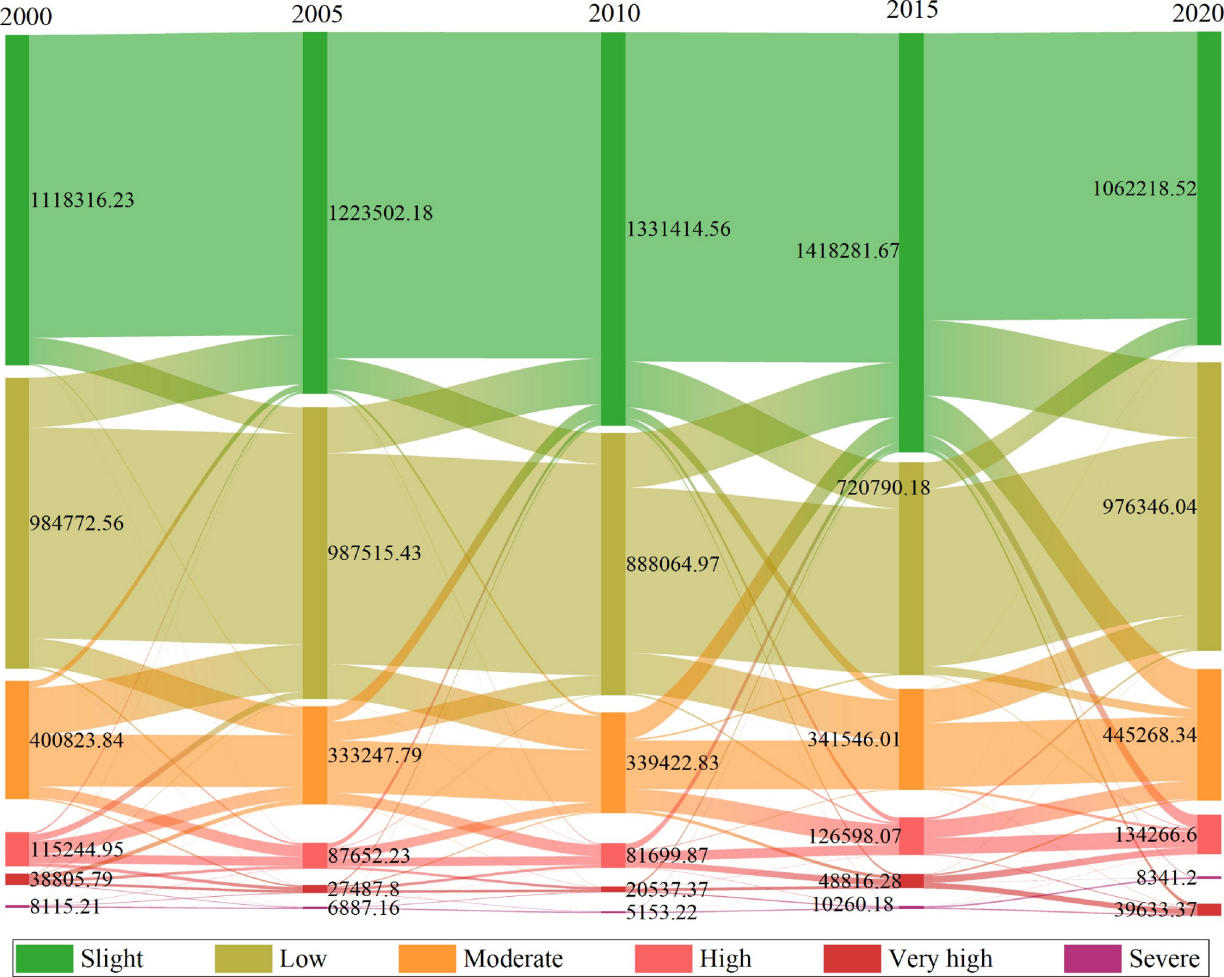
Dynamics of soil erosion intensity in Bijie over the past 20 years (ha).

This study computed land area changes under various erosion intensities in Bijie between 2000 and 2020 to examine the time-series change characteristics of soil erosion intensity (Fig 6). In Bijie, the predominant levels of soil erosion were low and slight, with the mildly eroded area reaching its peak in 2015, followed by an initial upward trend. The change in land area with low erosion was more volatile, experiencing an increase followed by a decrease, and then an increase again, reaching its lowest point in 2015 with an area of only 720,790.18 ha. The land area change trend in moderate erosion intensity decreased and then increased, with most of the decrease shifting to low erosion intensity. At high intensities, there was a comparable trend in the decline and subsequent increase in soil erosion. These results suggest that techniques for conserving soil and water can effectively improve soil erosion conditions; however, over time, these effects require further monitoring and management to ensure long-term sustainability.

### Factors affecting spatiotemporal variability of soil erosion

**Fig 7:**
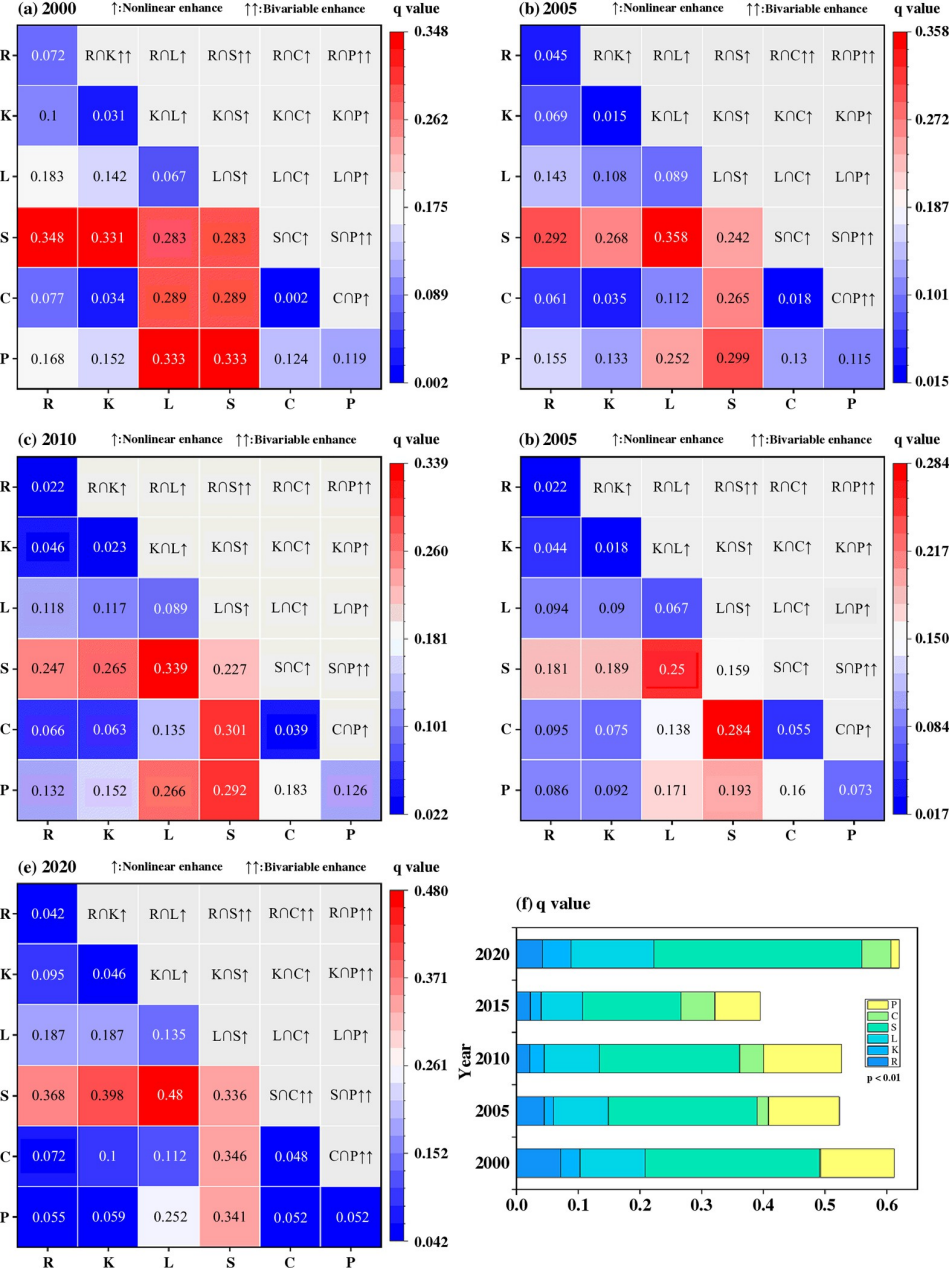
Interaction factor and q-value detection of the SE in the study area.

Fig 7 illustrates the geographic distribution of soil erosion in Bijie over the past 20 years, with the *P* values for all components being considerably less than 0.001. The factor contributions vary slightly from year to year. In 2000, the three factors with the most significant influence on soil erosion were *S*, *P*, and *L*. By 2020, the three most influential factors were *S*, *P*, and *C*. The slope factor had the largest impact on soil erosion across the research period, with q values from 2000 to 2020 of 0.283, 0.242, 0.227, 0.159, and 0.336. Geographical variations in soil erosion can be better explained by the interaction of multiple factors than by any factor acting alone [36]. In addition, the results in Fig 7 indicate that the main type of interaction is nonlinear enhancement, particularly the interactions between *P* and *R*, as well as *S* and *C*. In summary, rainfall, slope, and land use in Bijie were the main factors causing soil erosion.

## Discussion

### Effect of different slope zones on soil erosion

Geographical detector calculations showed that slope is a major element affecting soil erosion; thus, to improve our understanding of how slope influences soil erosion, we analyzed the soil erosion characteristics under different slope types. The slope intervals were divided into five categories: >5°, 5–8°, 8–15°, 15–25°, and >25°. Within the less than 5° interval, the area occupied by slight erosion was most significant. For the 8–15° slope interval, slight and mild erosion dominated. Moderate erosion occupied the largest percentage of the area in the 15–25° range. In contrast, in the zone with slopes exceeding 25°, high, very high, and severe erosion types became the dominant erosion patterns. This trend clearly demonstrates that soil erosion rates were enhanced by increasing slopes, supporting the strong positive association between soil erosion and slope gradient.

**Fig 8:**
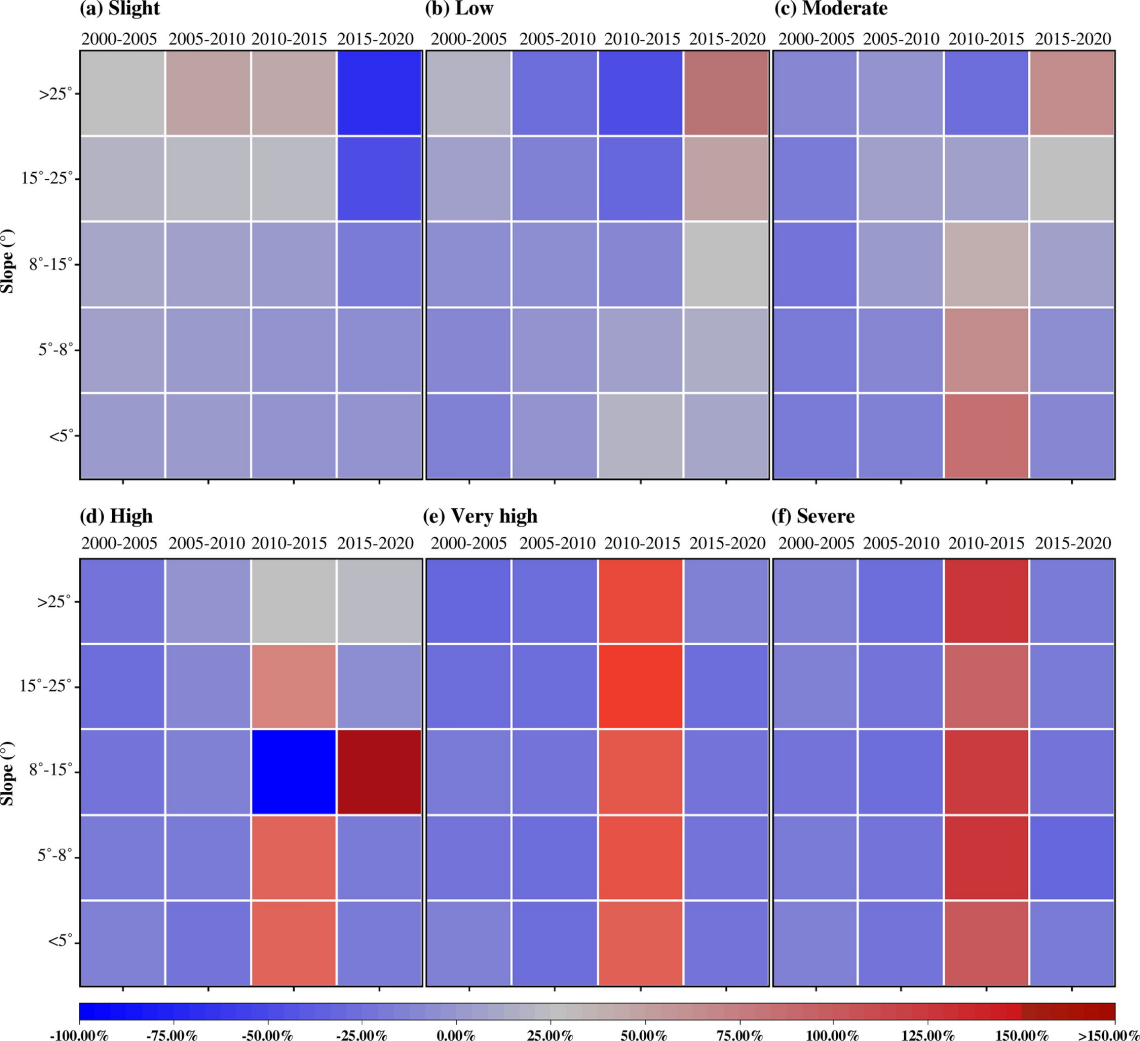
Rate of change of soil erosion area in different slope zones.

To characterize the changes in the area of soil erosion on different slopes, the rate of change in the erosion area in the five types of slope zones was determined, as illustrated in Fig 8. For slopes less than 8°, areas of slight and mild soil erosion showed little fluctuation between 2000 and 2020. The areas of moderate-to-severe soil erosion first decreased between 2000 and 2010, then increased in 2015, and then decreased again in 2020. For terrain with slopes between 8° and 15°, the trend in the area of different erosion types was similar to that of terrain with smaller slopes, with a significant decrease in areas of high erosion between 2010 and 2015, followed by a sharp increase in 2020. The area of slight erosion increased on terrain with slopes between 15° and 25° from 2000 to 2015, after which it significantly decreased. Mild erosion increased until 2005, decreased until 2015, and increased sharply thereafter. Moderate erosion typically decreases first and then increases. Erosion categories that were high and above demonstrated an increase, followed by a decrease. For inclines greater than 25°, gentle to moderate soil erosion displayed a comparable pattern, characterized by an increase in the area affected by erosion followed by a significant decline, whereas the area of high-intensity erosion decreased from 2000 to 2010 and increased slightly by 2020. Both very high and severe soil erosion rates decreased between 2000 and 2010, then increased significantly between 2010 and 2015, but began to decline again between 2015 and 2020. This could be attributed to the predominance of forest land in the higher-altitude areas of Bijie, where the ecosystem is relatively stable, decreasing the rate of soil erosion. However, lower slope regions are more vulnerable to human impacts.

### Applicability of soil erosion models

To select the RUSLE factors, this study drew on similar research results to increase the rationality of the model. Precipitation erosive power is the primary factor that determines soil erosion modeling [65]. This study collected the monthly average rainfall data of Bijie; thus, more variability can be observed on a monthly scale. Empirical values have been used as indicators of C [66]; however, this approach may not be fully compatible with the specifics of vegetation change. Therefore, annual NDVI data were used to ensure temporal consistency of the vegetation cover data.

The soil erosion modulus in Bijie showed a decreasing and then an increasing trend during the study period, which could be attributed to its rapid urbanization, increased population density, and homogenization of the land-use structure. This pattern illustrates the impact of the initial rocky desertification-mitigation initiative. However, the increase in the later data was closely related to the increased erosive power of rainfall, particularly the regular occurrence of intense rainstorms. Intense rainfall is a critical factor affecting the erosion process, with soil erosion being particularly pronounced during periods of extreme precipitation. Studies have shown that the risk of erosion increases significantly when rainfall erosive power maxima coincide with bare soil [67,68]. The final calculation gave an average erosion modulus for the study area of 10.04–13.45 t·ha^−1^·a^−1^, an outcome that is comparable to research conducted in other nearby karst zone regions [12,58]. From 2000 to 2020, the majority of the soil erosion in the study area was mild to moderate, representing over 76% of the total erosion. This observation aligns closely with findings reported in the Guizhou Province Soil and Water Conservation Bulletin.

### Limitations and significance of this study

This study employed the classic RUSLE model with factor parameters adjusted to account for karst geomorphological features because soil erosion is a long-term evolutionary process. Rock exposure is an important factor influencing soil erosion in karst environments [69], and more research is required to thoroughly examine the precise impact of rock exposure. Furthermore, when calculating soil erosion factor K, difficulties were encountered in the collection of local soil erosion coefficient measurements; future studies should refine the soil erosion coefficient using experimental measurement methods. Despite these unavoidable constraints, this study clearly and comprehensively reveals the spatiotemporal distribution characteristics of soil erosion over the past 20 years. The findings of this study have important applications for creating and improving plans to stop and manage soil erosion, as well as policies in karst plateau mountainous regions. In addition, field studies [70] can be conducted in areas experiencing significant changes in soil erosion to assess the impact of local human activities and propose specific measures for mitigation.

## Conclusions

This study used RUSLE to statistically analyze the spatiotemporal aspects of soil erosion in Bijie, a mountainous region in the Karst Plateau, over the past 20 years. A geographic detector was used to investigate the factors impacting soil erosion. The results indicate that soil erosion tended to decrease between 2000 and 2020, followed by an increase. The regions of southern, northern, and southwestern Bijie are characterized by substantial erosion. The degree of land use, slope gradient, and precipitation were the main factors affecting soil erosion in Bijie, with slopes having the greatest impact. These findings explain the spatiotemporal evolution of soil erosion in karst plateau mountainous areas, as well as the main factors influencing soil erosion, providing theoretical guidance and technical support for understanding the law of soil erosion and improving soil erosion prevention and control systems. At varying slope levels, it is advisable to implement appropriate preventive and control methods for soil erosion, such as establishing a vegetation protection system, improving land utilization, and strengthening the construction of governance projects, to lessen the detrimental effects of soil erosion on agricultural output and the natural environment. Simultaneously, strengthening monitoring and investigation to obtain timely, thorough, and precise knowledge of soil erosion and associated conditions is essential for the scientific development of prevention and control strategies, enhancement of early warning and emergency response systems, and mitigation of soil erosion losses.
